# Non-pharmacological therapeutic paradigms in stress-induced depression: from novel therapeutic perspective with focus on cell-based strategies

**DOI:** 10.1017/neu.2024.39

**Published:** 2025-03-20

**Authors:** Maryam Azarfarin, Mahdi Mohammadzadeh Shahla, Gisou Mohaddes, Masoomeh Dadkhah

**Affiliations:** 1 Neuroscience Research center, Tabriz University of Medical Sciences, Tabriz, Iran; 2 Student Research Committee, Tabriz University of Medical Sciences, Tabriz, Iran; 3 Department of Neuroscience, Faculty of Advanced Medicine, Tabriz University of Medical Sciences, Tabriz, Iran; 4 Student Research Committee, Ardabil University of Medical Sciences, Ardabil, Iran; 5 Department of Biomedical Education, College of Osteopathic Medicine, California Health Sciences University, Clovis, CA, USA; 6 Pharmaceutical Sciences Research Center, Ardabil University of Medical Sciences, Ardabil, Iran; 7 Neuroscience Research Group, Pharmaceutical Sciences Research Center, Ardabil University of Medical Sciences, Ardabil, Iran

**Keywords:** depression, stress, therapy, cell-based strategies, non-pharmacological paradigms

## Abstract

Major depressive disorder (MDD) is considered a psychiatric disorder and have a relationship with stressful events. Although the common therapeutic approaches against MDD are diverse, a large number of patients do not present an adequate response to antidepressant treatments. On the other hand, effective non-pharmacological treatments for MDD and their tolerability are addressed. Several affective treatments for MDD are used but non-pharmacological strategies for decreasing the common depression-related drugs side effects have been focused recently. However, the potential of extracellular vesicles (EVs) derived from mesenchymal stem cells (MSCs), microRNAs (miRNAs) as cell-based therapeutic paradigms, besides other non-pharmacological strategies including mitochondrial transfer, plasma, transcranial direct current stimulation (tDCS), transcranial magnetic stimulation (TMS), and exercise therapy needs to further study. This review explores the therapeutic potential of cell-based therapeutic non-pharmacological paradigms for MDD treatment. In addition, plasma therapy, mitotherapy, and exercise therapy in several in vitro and in vivo conditions in experimental disease models along with tDCS and TMS will be discussed as novel non-pharmacological promising therapeutic approaches.

Highlights

1. Major depressive disorder (MDD) is considered a psychiatric disorder and have a relationship with stressful events.
2. Cell-based therapeutic paradigms along with exercise, plasma, and mothotherapy are well-stablished non-pharmacological therapies for depression.
3. MicroRNAs expression in exosomes are potential biomarker candidates for the pathogenesis of central nervous system-related diseases.

## Considerations

Although the current review is not based on fully comprehensive systematic analysis of all publications on recent methods reported. Thus, a wide range of the studies referenced, are based on data obtained from relatively limited number of preclinical studies and clinical studies. Nevertheless, the mentioned approaches discussed above can provide new advances in treatment and can be more effective methods for treating depression. By improving future advances and characterisation of EV-based, mesenchymal stem cell (MSCs)-based, mitotherapy, and plasma therapy strategies, relevant advances in depression therapy may shed light on defining novel approaches on depression therapy. Whether this rather non-pharmacological may be sufficient to alleviate the depression-related alternatins reported in depression therapy needs to be further assessed.

## Summations

1. This review summarises the evidence how depression as a psychiatric disease associated with stress and highlights the protective effects of exosome-based therapy, MSC therapy, and mitotherapy against chronic stress-induced depression, which could alleviate stress-induced symptoms. These methods are considered novel promising therapeutic strategies for depressive disorders. Two key factors in stress-induced depression are neuroinflammation and hypothalamic–pituitary–adrenal (HPA) axis hyperactivity.
2. Identifying the possible relationship between stress and psychiatric disease opens new non-pharmacological strategies to alleviate depression symptoms.
3. MicroRNAs with high specificity and sensitivity in diagnosing depression are important candidates for depression diagnostic biomarkers.
4. Cell-based therapeutic paradigms, mitotherapy, and plasma therapy can be suggested as novel non-pharmacological promising therapeutic approaches.

## Limitations

We have done a complete search to collect a comprehensive review until December 2023. The current paper aims to review the non-pharmacological approaches to the treatment of MDD, but is not a systematic review. The references reviewed are mainly include research articles and systematic reviews, but there may be related studies that may not be discussed here. For these reasons, the paper does, not listed recommendations for disease treatment. Nonetheless, it should help to response precisely to more questions related to the development of the novel therapeutics for MDD in the near future.

Due to the limited number of preclinical studies in cell-related therapies through BMMCs or MSCs transplantation as well as the administration of exosomes as well-known cell products, may have a place in TRD. However; further clinical trials are encouraged to be developed.

Mitochondrial transfer from one cell to another has potential benefits, but the use of this method by cell-to-cell fusion remains still in the early stages of research, and additional researches are needed to fully explore its potential and limitations (Clemente-Suárez et al., 2023)

Also, the transfer of mitochondria mediated by exosomes also has some limitations. One of the major issues is the efficacy for the transfer of damaged mitochondria. Exosomes that are secreted by cells under stress status or in diseased conditions may contain dysfunctional mitochondria, which could exert as negative impact on the recipient cell’s metabolism (O’Brien *et al.*, 2020).

Despite its potential benefits, mitochondrial transfer also has several limitations. Transferred mitochondria contain genetic material and this major concern results in immune rejection of the transferred mitochondria (Babajani *et al.*, 2021) raised from oocyte transfer, as well as stem cell therapy. Another concern is the risk of introducing mitochondrial mutations, which can make some unpredictable outcomes. Further research is needed to address These concerns need to further research to develop safe and more effective transfer approaches (Smeets, 2013). However, limitations in drug delivery and specificity have hindered the success of these therapies (Manzari *et al.*, 2021). Therefore, there is a critical need for novel Mitochondrial-targeted therapies.

Further studies should also focus on other preventive approaches with recently highlighted ameliorating depressive symptoms through effect on structure and function of brain areas or even dysfunction of the HPA axis, which were not mentioned in this review.

## Introduction

Major depressive disorder (MDD) is the most common cause of neuropsychiatric diseases in the world (Freidrich, 2017) and mainly co-exists with anxiety (Hirschfeld, 2001), which contributes to ∼85–90% of the total population who are suffering from these debilitating conditions (Tiller, 2013). This mood disorder has a high mortality and recurrence rate. Due to the high mortality rate of depression (Reddy, 2010), many patients with depression suffer from sleep disorders and symptoms like anorexia, anxiety, and gangrene and are prone to suicide (Hammen, 2005).

Chronic stress involves in the aetiology of mood disorders including anxiety and depression as one of the most crucial environmental factors (Bozorgi *et al*., 2024). Stress is a common response to threatening stimuli results in physiological alternations, which are critical for survival. Sustained stress condition, in contrast, have a role in emergence of a wide variety of neuropsychiatric disorders. Chronic stress-induced anxiety disorders have caused significant public health concerns recently. Anxiety is among the most common mental disorders, which is highly prevalent globally (Azarfarin *et al*., 2018). Individuals exposed to chronic stress present heightened vulnerability to anxiety, depression, and stress-related mood disorders (Seo *et al*., 2017). Furthermore, the development of the different therapeutic options against neuropsychiatric disorder, such as anxiety and depression, is an interesting topic for researchers (Majidi-Zolbanin *et al*., 2013). A broad categorisation of treatments for depression including pharmacological interventions, non-pharmacological approaches, or a combination of both have been documented (National Institute for Clinical Excellence %J Clinical guidelines, 2009).

Despite administering of the common pharmacological antidepressant drugs as first-line treatment in MDD, many patients are treatment-resistant. Thus, different studies have been carried out on novel therapeutic approaches, especially non-pharmacological strategies in treating MDD. In addition, clinical studies have represent that prescribe of antidepressants to older patients results in increased adverse drug-related events risk and probable drug interactions with other medications (Everitt *et al*., 2018). Non-pharmacological therapies; however, are effective in relieving the depression symptoms among older depressed adults (Apóstolo *et al*., 2016, Holvast *et al*., 2017, Baba *et al*., 2022). These therapies include a wide range of approaches, such as cell-based therapies, brain stimulation therapies [(e.g. transcranial direct current stimulation therapy and transcranial magnetic stimulation (TMS)], and non-invasive therapies plasma therapy, an exercise therapy (Gertler *et al*., 2015, Javani *et al*., 2023).

Among non-pharmacological therapies, exosomes have received much more attention due to their efficacy in acupuncturing of several diseases. Furthermore, they are heterogeneous and targeted, and their rate, size, and composition are highly dependent on the parental cell (Zhang *et al*., 2015) . In particular, the composition has specific proteins, lipids, mRNA, and microRNAs (miRNAs), all of which are associated with the parental cell. Recently, several studies showed that exosomes are involved in pathological processes, such as neurogenesis (Wei *et al*., 2020), neuroinflammation (Brites and Fernandes 2015), and other pathological processes. Current modern biological studies proposed that exosomes can be categorised as a source of depression-specific markers for the diagnosis and treatment of MDD (Lyu *et al*., 2023).

A wide variety of studies also refers to exercise role in ameliorating stress-induced anxiety and depressive-like symptoms (Rashidi *et al*., 2017, Moradi-Kor *et al*., 2020). Exercise has been shown an effective alternative option to drugs and psychotherapy (Recchia *et al*., 2022). Exercise, also exerts an impact on range of physical and cognitive outcomes (Owen *et al*., 2020).

Recent studies have provided evidence that mitochondria-mediated mechanisms are associated with depressive symptoms among neurobiological and psychological theories explaining the pathogenesis of depression 2. At present, mitochondrial transplantation is receiving increasing attention within the context of treatment for mood disorders. Mitochondria can be transferred into damaged cells with the use of a variety of methods and can be easily incorporated and tracked inside host cells 9 (Javani *et al*., 2023). In mice model of depression, intravenous injection of exogenous mitochondria resulted in decrease depressive behaviour, reduced activation of microglia and astrocytes, the reduction of inflammatory processes, and the increase of neurogenesis in the hippocampus.10. While neurobiological and psychological theories explain the pathogenesis of depression, several studies focused on mitochondria-mediated mechanisms which are related to depressive symptoms, however, considered the therapeutic techniques targeting mitochondrial (Głombik *et al*., 2021).

Morever, plasma therapy can partially improve the neuropathology of depression symptoms through mechanisms such as altering the apoptotic signalling pathways (Ghaffari-Nasab *et al*., 2021). Importantly, numerous researches have suggested that treatment with exercise should be administered only clear signs of depression. This paper aims to explore various non-pharmacological interventions (i.e. cell-based therapies, exercise, plasma, transcranial direct current stimulation (tDCS), and TMS therapy based on preclinical and clinical studies highlighting the critical role of cell-based therapies, in depression status and the treatment of MDD. Also, this review aims to discusse the potential efficacy and provide the scene for a future approaches on the specific aspects of MDD non-pharmacological therapies with respect to the related mechanism.

## Methods

In the current review study, the five main mega databases, including PubMed, Google Scholar, Scopus, Springer, and Science Direct, were ultimately conducted by three researchers who searched using ‘non-pharmacological’, ‘neuropsychiatric disorders’, ‘exosomes’, ‘miRNAs’, ’stem cell therapy’, ‘plasma therapy’, an ‘exercise therapy’, ‘transcranial direct current stimulation therapy’, and ‘transcranial magnetic stimulation’ as our selected keywords. All related articles were collected from 2001 to 2023, and all relevant articles were finally enrolled.

### Pathophysiology of depression

#### Genetics

Although identifying the role of individual genes responsible for depression is challenging, a number of MDD risk loci have been reported (Zeng *et al*., 2017, Zeng *et al*., 2017). In a large cohort study on MDD patients, Hyde et al., (Hyde *et al*., 2016) discovered 15 genetic loci associated with MDD risk in 2016. In another independent study, Wray *et al*., (Wray *et al*., 2018) founded 44 risk loci through a meta-analysis study on MDD so far. In addition, a recent genome-wide association study (GWAS) reported 102 independent variants related to depression (Howard *et al*., 2019). Generally, all this evidence suggests that genetic factors influenced the MDD.

#### Neurotransmitter systems

The critical role of neurotransmitters is well documented in depression aetiology (Kong *et al*., 2021). For example, due to the wide distribution of serotonin (5-HT) in the nervous system, deficiency in this neurotransmitter results in depression (De-Miguel *et al*., 2005). Three possible mechanisms have been proposed for impaired 5-HT1A function in depression, including social isolation reducing 5-HT1 neurotransmission, 5- HT2 receptors inhibiting 5-HT1 neurotransmission, and hypercortisolaemia inhibiting 5-HT1 neurotransmission (Deakin and Graeff 1991). In addition, endogenous agents, such as brain-derived neurotrophic factor (BDNF) and neurotrophin-3, are associated with the growth and function of serotonergic neurons in the adults brain (Xue *et al*., 2021).

Furthermore, dopamine (DA) is a precursor to epinephrine and norepinephrine (NE) and acts as a dominant transmitter in the brain, regulating behaviour (Babaev *et al*., 2022). Various human and preclinical studies have reported that depression and DA transmission are the main players in the central nervous system (CNS) (Salamone *et al*., 2022). Additionally, depressed patients showed elevated levels of DA transport (Duval *et al*., 2021), which leads to more effective reuptake of DA in presynaptic neurons.

A large body of evidence suggests that depression is linked to the glutamate system (Chen *et al*., 2022), since elevated levels of glutamate were observed in the blood, cerebrospinal fluid (CSF), and brains of patients affected by depression (Hashimoto and Psychiatry, 2011). Patients with depression also showed disturbances in the N-methyl-D-aspartate receptor (NMDAR) subunit in the brain (Chandley *et al*., 2014). Finally, γ-aminobutyric acid (GABA) as an inhibitory neurotransmitter is present in a small percentage in neurons in contrast to glutamate (Duman *et al*., 2019). GABA-ergic neurons are widely distributed in the brain and participate in various functions, including the regulation of anxiety, motivation, and the reward system (Lowes *et al*., 2021, Zhang, Liu *et al*., 2021). GABA has a critical role in alleviating MDD symptoms (Petty *et al*., 1995). In this regard, based on numerous human studies, the GABA neurotransmission function MDD patients have defects in GABA neurotransmission function (Fee *et al*., 2017, Ghosal *et al*., 2017).

#### Neuroinflammation

Elevated pro-inflammatory peripheral biomarkers and immune dysfunction-related diseases are high risk factors for depression. Furthermore, the ability of immune mediators to induce depressive symptoms and the ability of activated microglial cells to decrease serotonin levels, all demonstrated the immune system’s role in the pathogenesis of depression (Nettis and Pariante, 2020). Cytokines, especially interlukine-6 (IL-6), are important markers for inflamed depression and further direct treatment (Lynall *et al*., 2020). Thus, the elevation of IL-6 may be involved in long-lasting inflammation and other pathological processes in depression (Mac Giollabhui *et al*., 2021).

#### Mitochondrial dysfunction

Mitochondria are subcellular organelles with a critical role in various functions, from producing energy and involvement in cell signalling to hormone production and metabolism. This organ depends on energy and densely populates the brain (Juster, Russell *et al*., 2016). One of the common features of many diseases is associated with mitochondrial dysfunction. Mitochondrial dysfunction profoundly affects psychological processes, and there is a strong link between stress-induced mitochondrial dysfunction and psychopathology (Tyrka *et al*., 2016, Trumpff *et al*., 2019). Mitochondrial disorders also contribute to affective alternations, so mitochondrial dysfunction expresses comorbid psychiatric problems in more than half of the depressed patients (Kasahara and Kato, 2018). Furthermore, mitochondrial dysfunction is observed in neuropsychiatric disorders such as Alzheimer’s and Parkinson’s diseases (Yan *et al*., 2013, Flannery and Trushina, 2019). The stress response is an energy-required physiological process that allows the organism to adapt against this challenge, increasing the availability of energy substrates, such as glucose, especially in the brain (Magistretti and Allaman, 2015). The required energy for key enzymatic reactions, transcription and translation of genes, release and reuptake of neurotransmitters, the production of hormones, sympathetic activation, and behavioural adaptations is provided by mitochondria (Picard and McEwen, 2018). Stress exposure mediators accelerate the mitochondrial release of mitokines as signalling molecules. These molecules serve as signals and include different mitochondrial metabolites, calcium, and reactive oxygen species (ROS) (Shaughnessy *et al*., 2014, Chandel, 2015). ROS are generated within mitochondria at low levels as a byproduct of the energy-producing processes, supporting several critical functions in the cell (Meyer *et al*., 2018). As discussed, mitochondria potentially activate the stress-response system to cause alternations in brain morphology and capabilities. Increased energy needs from the stress-response system produce increased ROS in mitochondria, suppressing cells’ antioxidant capacity, contributing to mitochondrial DNA mutations (Lagouge and Larsson, 2013).

#### Hypothalamic–pituitary–adrenal axis

Stress, as a contributing factor in MDD onset (Tan *et al*., 2021) has an effect on the *hypothalamic–pituitary–adrenal* (HPA) axis and plays a fundamental role in stress response. However, any change in the HPA axis during depression may reflect the influence of stress in presenting depressive symptoms. Stress triggers the release of corticotropin-releasing hormone (CRH) through stimulating adrenocorticotrophic hormone (ACTH) secretion in the pituitary gland, which subsequently increases glucocorticoid production from the adrenal cortex (Sukhareva and Breeding 2021). The HPA axis is overactive under stressful conditions, resulting in major problems in depressed patients such as hypercortisolemia, decreased rhythmicity, and higher cortisol levels (Pariante and Lightman 2008). Dysfunction in the HPA axis induced by stress has been shown to be associated with depression as a result of increased production of cortisol and disturbances in suppressing the glucocorticoid receptor regulatory feedback (Keller *et al*., 2017).

#### Neurotrophins and neurogenesis

The neurotrophin hypothesis of depression comes from primarily the theory that lowered hippocampal BDNF levels are related to stress-induced depression (Li, Shen *et al*., 2018). Mounting evidence identifies that BDNF levels are decreased in the postmortem peripheral blood of individuals with depression (Dwivedi, 2009). Moreover, BDNF depletion seems to impair neurogenesis and be involved in the onset of MDD. It is targeted by antidepressant agents, which could mitigate MDD symptoms by elevating brain BDNF levels.

#### Metabolic disorders

**Figure 1.**
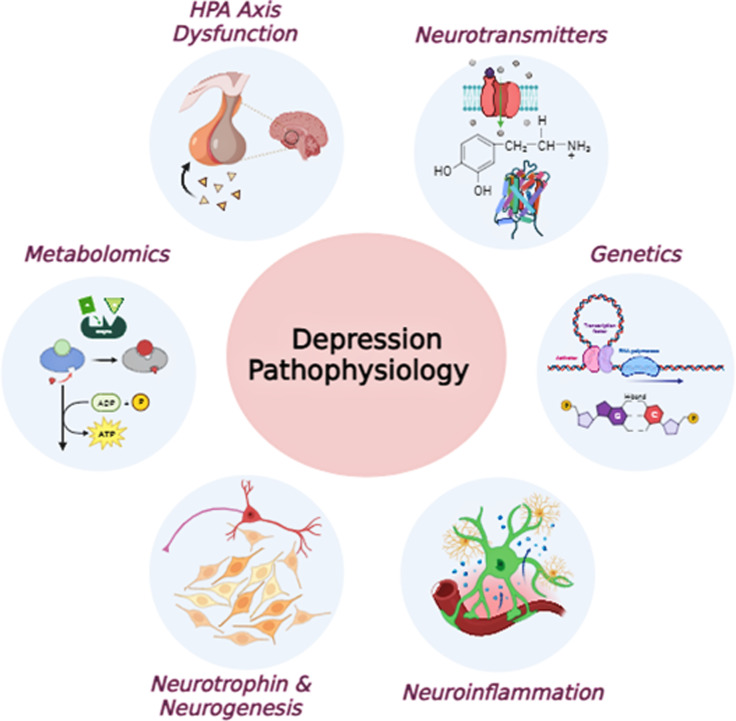
Pathophysiology of depression. HPA: hypothalamic–pituitary–adrenal axis.

Patients with metabolic disorders often suffer from depression and experience it throughout their lives (Duarte-Silva *et al*., 2021). Data from experimental studies on animal models and clinical practice showed that metabolomics contribute to investigating the pathophysiology of depression and can be used as a potential biomarker. These compounds have been identified as effective agents for selecting related animal models for depression research (Shi *et al*., 2013). For example, (Zheng *et al*., 2013) observed differentially expressed metabolites in MDD subjects compared to healthy control cases and reported five metabolites as strong biomarkers for MDD (Figure 1) (Zheng *et al*., 2013).

### Chronic stress, neuroinflammation, and depression

Chronic stress results in hyperactivity in immunological processes, demonstrating a potential pathway underlying depression. The innate and adaptive immune responses disrupt the integrity of the blood–brain barrier (BBB), resulting in the development of inflammatory signals directionally between the periphery and CNS (Medina-Rodriguez and Beurel, 2022). Mounting evidence supports a positive link between the elevated concentration of inflammatory cytokines in serum and CSF and depression severity and treatment resistance (Osimo *et al*., 2019). Moreover, stress exposure appears to be a melancholic phenotype of depression, induces higher insulin insensitivity, and increases pro-inflammatory cytokines (10). Neuroinflammation, which refers to the central innate immune system activation, leads to a depressive phenotype that occurs through severe symptomatology with higher morbidity and mortality (Maes *et al*., 2012). Although there is a lack of unifying diagnostic criteria for inflammation in depression, a peripheral C-reactive protein (CRP) level is a common marker of neuroinflammation (8). Due to the strong link between peripheral and CRP levels in CSF, it could be a logical marker of neuroinflammation (9). Increased inflammatory cytokines in plasma, most consistently interleukin IL-1β, IL-6, and tumour necrosis factor (TNF-α), contribute to the appearance of depressive symptoms since these cytokines sensitise the HPA axis (pituitary) activity, disturb the negative feedback loop, and result in developed inflammatory reactions. Neuroinflammation exacerbates through peripheral inflammation via a number of mechanisms, including disrupted BBB, cellular immune trafficking, and induction of glial cell activation (51–16). According to preclinical studies, there is a well-known relationship between cortisol level and depression in which dysregulation in cortisol level is correlated with depressive-like behaviours. For example, social avoidance behaviour was induced following the deletion of the glucocorticoid receptor nuclear receptor subfamily 3 group C member 1(NR3C1) gene in astrocytes, and this deletion downregulates adenosine triphosphate (ATP) release through the phosphoinositide 3-kinase (PI3K)/protein kinase B (AKT) (PI3K-AKT) signalling pathway (13), supporting glucocorticoid signalling involvement in response to stress, inflammation, and consequent depression (15).

### Brain circuits involved in depression and stress-induced dysfunction

Across recent decades, progress in human and animal-based methods has significantly developed our understanding of the underlying neural mechanisms associated with psychiatric disorders. To provide insight into the involvement of specific brain regions in the aetiology and treatment of depression, a broad range of experimental methods have been adopted, from basic studies to more clinically relevant animal models. Additionally, dysfunction of prefrontal subregions and circuits determines the dysregulation in depression (Ferenczi *et al*., 2016, Alexander *et al*., 2019). Chronic stress is considered a potential risk factor for a wide range of neuropsychiatric conditions, like depression. Considering the profound effects of stress as a well-known cause of depression (Duman and Li, 2012), the function and morphology of the hippocampus, amygdala, and prefrontal cortex (PFC) appear to be especially dysregulated (Cook and Wellman, 2004, Moradi-Kor *et al*., 2019, Ghorbanpour *et al*., 2021).

Neuroimaging studies have delineated disturbances in the limbic structure, including the amygdala, PFC, anterior cingulate cortex, and hippocampus, involved in the neuropsychiatric conditions’ pathophysiology (Etkin *et al*., 2009). Alternations in brain neural circuits or a chemical imbalance in the brain contribute to the expression of depression (Palazidou, 2012). Advances in functional brain imaging as a noninvasive method suggest that dysfunctions in several brain circuits can underlie the onset of depression and anxiety disorders (Goldstein-Piekarski *et al*., 2022). Data obtained from structural magnetic resonance imaging (MRI) research demonstrated that depression causes reductions in the thalamus, basal ganglia, hippocampus, PFC, and orbitofrontal cortex volume, as well as in the amygdala and anterior cingulate cortex (Kempton *et al*., 2011). Stressed conditions affect its activity, indicating the hippocampus’s involvement in stress-induced depression (Krugers *et al*., 2010).

### Potential biomarkers for depression

The term ‘diagnostic biomarkers’ is often used to describe characteristics of markers that are useful in determining the presence or absence of a disease state, and the term ‘therapeutic biomarkers’ is used to predict treatment response (Perlis, 2011).

Evidance obtained from markers reflecting the activity of the inflammatory, metabolic, neuroendocrine, neurotransmitter, and neurotrophic systems may be able to predict physical and mental health outcomes in depressed patients; however, there are many discrepancies in the results (Jani *et al*., 2015). Early biomarker research was concentrated on blood-detectable neurotransmitter metabolites. In the clinic, using serotonin reuptake inhibitors suggested that the serotonergic system was involved in depressive disorders, so early biomarker investigations focused on measuring serotonin levels and its main metabolite, 5-hydroxyindoleacetic acid (Ritsner, 2009). During the 1970s and 1980s, the dexamethasone cortisol suppression test was recommended as a highly promising method (Green and Kane, 1983).

Small proteins, known as cytokines, are involved in cellular signalling networks. Different cells in the body produce a superfamily of cytokines. Even though most cytokines are secreted, some are also expressed on cell membranes, and others are retained in the extracellular matrix (Fitzgerald, 2001). The effects of IL-1 receptor antagonists, interleukin-1 beta (IL-1β), IL-6, interferon-gamma (IFN-γ), or TNF-α, have been supported by various studies (Simpson *et al*., 1997, Dantzer *et al*., 1999, Licinio and Wong, 1999; Tsai, 2017). Recently, more cytokines, their receptors, and ligands have been investigated for mood deterioration and the risk of depression development (Lotrich, 2015). The relationship between neuroinflammation and T helper 17 (Th-17) cells with IL-17A is being investigated (Beurel and Lowell, 2018). Systematic reviews and meta-analyses also demonstrated that levels of IL-1β, IL-2, IL-4, IL-5, the soluble IL-6 receptor (sIL-6R), IL-8, IL-17A, transforming growth factor-β (TGF-β1), and CCL3 are substantially altered in depression (Köhler *et al*., 2017, Byrne *et al*., 2020, Liu *et al*., 2020, Neupane *et al*., 2022, Yui *et al*., 2022).

In addition, CRP is one of the known inflammatory biomarkers in many patients with depression (Uher *et al*., 2014). Anti-cardiolipin antibody (aCL) as an inflammatory marker may also be a suitable candidate. Patients with MDD exhibited high plasma titres of aCL immunoglobulin M (IgM) that were noticeably greater than those of healthy control participants (Costa *et al*., 2022). Although this research did not show any association between changes in depression symptoms and alterations in aCL IgM titres, it did show that aCL IgM titres changed substantially during a 12-week antidepressant therapy (Serretti, 2022).

BDNF is classified as a family of growth factors, including the nerve growth factor, neurotrophin-4/5, and neurotrophin-3 (de Assis and de Almondes, 2017). BDNF is a small molecular dimeric protein that facilitates the growth and neural differentiation of neurons in the CNS. The key role of BDNF and its high affinity receptor, tropomyosin receptor kinase B (TrkB), has been established in the pathophysiology of depression (Björkholm and Monteggia, 2016). Numerous investigations have shown that depressed individuals’ serum levels of BDNF are lower than those of healthy people (Molendijk *et al*., 2014), and a considerable rise in BDNF levels was seen after antidepressant treatment. Additionally, it was shown that BDNF levels in the serum increased during therapy in patients with severe mental illness, although they did not fully recover (Nuernberg *et al*., 2016). Also, in the samples obtained from depressed patients brain, compared to patients who did not take antidepressants, those who took antidepressants had higher levels of BDNF expression in the hippocampus (Chen *et al*., 2001).

Increasing our knowledge about the function of BDNF in the pathophysiology of depression will aid in developing and manufacturing several more beneficial pharmacological drugs for treating depressive disorders. People with mental illnesses often have aberrant lipid levels (Hamilton *et al*., 2007). It has been shown that there is a high prevalence of lipid and glucose abnormalities in patients with schizophrenia and mood disorders (Wysokiński *et al*., 2015). Depression and suicidality have been linked with elevated blood triglyceride levels. Additionally, a strong relationship exists between depression in women and high triglyceride levels (150 mg/dL) (Oh and Kim, 2017). Patients with a first episode of MDD had lower high-density lipoprotein (HDL) levels (Wei *et al*., 2020). Low HDL has also been shown to be associated with an immunometabolic subtype of depression (Alshehri *et al*., 2023). Moreover, decreased serum HDL and increased urinary 3-NT, considered together, can strongly indicate depression (Nobis et al., 2023). The research supports the significance of oxidative stress and abnormalities in cholesterol in MDD. More research is needed to evaluate their clinical utility as markers. Despite all the challenges and limitations, biomarkers to improve patients’ lives worldwide seem likely to significantly impact the psychiatric clinic in the coming years.

### Non-pharmacological therapeutic features for depression

#### Exosomes in depression diagnosis and therapy

Exosomes are microvesicular bodies (MVB) with a unique disc- or cup-shaped morphology and 30–100 nm in size (Cocucci and Meldolesi, 2015). Exosomes belong to lipid vesicles, containing messenger RNAs, microRNAs, proteins, and liposomes, contributing to cell-to-cell communication and targeting cell flow out of bodily fluids, including blood, urine, CSF, and others (Xian *et al*., 2019). The composition of exosome miRNAs differs in patients with epilepsy, depression, and healthy individuals (Zhang *et al*., 2015).

They are composed of different clinically critical biomolecules, including proteins, lipids, nucleic acids, and even metabolites that demonstrate physiologically the status of the cell. In other words, the transfer of exosome content potentially affects intercellular communication in different physiological or pathological conditions (Yuyama and Igarashi, 2016). Neurotransmitters regulate exosome secretion, assisting communication between oligodendrocytes and neuronal cells. In this case, they can be crucial to neuronal myelination and integrity (Mitsis *et al*., 2020). Cargo molecules are transported bidirectionally via exosomes from the periphery to the brain and vice versa to pass the BBB. However, this ability makes them an attractive source of biomarkers originating from the CNS, which can be isolated peripherally from body fluids (Saeedi *et al*., 2019).

#### Exosomal microRNA profile in the serum of individuals with neuropsychiatric disorders

MicroRNAs, or miRNAs, are non-coding RNA molecules with a single chain of molecules and 18 to 24 nucleotides. In most cases, mature miRNAs control mRNA expression by binding to target mRNAs, destroying the mRNA, or repressing the translation (Bartel, 2009; Lu and Rothenberg, 2018). Although miRNAs comprise only 1 per cent of the human genome, they control 30 per cent of gene expression (Kwon *et al*., 2020). Numerous studies have shown that by controlling the stability or translation of various mRNA targets, miRNAs contribute to synaptic plasticity, which is linked to the development of MDD (Zhou *et al*., 2021). Additionally, miRNAs can enter the CNS after passing through the BBB and using exosomes as carriers to suppress various physiological functions, such as neurogenesis, neurotransmission, synaptic morphology, structure, and synaptic energy metabolism (Gao *et al*., 2022). Several investigations have shown a link between the amount of miRNA expression and the development of depression. The levels of expression of miRNAs in the amygdala, PFC, and other areas, as well as the characteristics and levels of their resulting target genes and proteins, were examined in human postmortem investigations (Al-Rawaf, Alghadir and Gabr 2021, He *et al*., 2021, Mizohata *et al*., 2021, Sundquist *et al*., 2021, Xian *et al*., 2022).

Numerous miRNAs, including miR15a, miR17-92, miR34, miR-101, miR-124, and miR-155, may be important in treating depression by regulating signalling pathways. It has also been indicated that the levels of peripheral miRNAs are susceptible to dysregulation, including the highly elevated levels of miR-124-3p and miR-451a and the downregulated levels of miR-320a and miR-335 (Hassan *et al*., 2022).

In a recent systematic review (Li *et al*., 2023), results indicated that microRNAs show high specificity and sensitivity in diagnosing depression, making them candidates for diagnostic biomarkers of depression. The blood plasma of 50 patients with depression and 41 healthy subjects was utilised to examine the changes and compare the levels of miRNAs in MDD patients (Camkurt *et al*., 2015). Comparing MDD patients with healthy people in the control group revealed substantial alterations in four miRNAs in quantitative polymerase chain reaction (qPCR) analysis. Of this, miR-320a was downregulated, whereas miR-17-5p, miR-223-3p, and miR-451a were upregulated. The authors suggested that miR-451a may be a potential biomarker for depression. Exosomal levels of miR-139-5p are much higher in MDD individuals (Liang *et al*., 2020). Patients with treatment-resistant depression (TRD) had considerably elevated levels of miR-335-5p and dramatically downregulated miR-1292-3p compared to healthy subjects (Li *et al*., 2021). In addition, the miR-30 group of miRNAs causes a depression-like phenotype induced by chronic stress through changing neuroplasticity and neurogenesis of the hippocampus through the control of transcription and epigenetic regulators (e.g. Runx1 and MIl 3) and also the regulators of cell signalling (e.g. Ppp3r1, Socs3, Nrp1, and Gpr125) (Khandelwal *et al*., 2019). Three miRNAs, including miR-16, miR-135a, and miR-1202, had remarkably lower serum levels in depressed patients who had spent at least two months without any medication (Gheysarzadeh *et al*., 2018). The tests, however, revealed no appreciable variation in the blood levels of miR-16 and miR135a in the patients before and after therapy with selective serotonin reuptake inhibitor (SSRI) antidepressants. However, miR135a levels in the blood of depressed patients after undergoing cognitive behavioural therapy (CBT) for 3 months revealed a significant increase (Issler *et al*., 2014).

The exosome extracted from the CSF with the content of miRNAs can pass the BBB; therefore, a biomarker representing depression can be derived from the peripheral blood (Reynolds and Mahajan, 2020). Besides, many studies indicate an abnormality in miRNA expression in depressed people. For example, animal and human studies introduce miRNA-124 as a biomarker for depression (Zobel *et al*., 2004). miRNAs expression in exosomes is widely altered in various disease conditions, making the miRNAs potential biomarker candidates for the pathogenesis of CNS-related diseases (Saeedi *et al*., 2019). Recently, researchers reported the possible contribution of exosomes and associated miRNAs to inflammatory mechanisms related to depression (Sakamoto *et al*., 2021). For example, the exosome-derived miR-139-5p might be a strong biomarker for MDD (Liang *et al*., 2020). These results support the participation of exosomes in the occurrence and development of depression and other mental deficits.

At last, miRNAs may be utilised to create novel medications, new biomarkers for the diagnosis of depression, or therapeutic targets. These advances can provide new and more effective methods for treating depression.

#### MSCs in depression diagnosis and therapy

MSCs are adult multipotent stromal cells with various sources. They can be isolated from bone marrow (BM-MSCs), adipose tissue (AD-MSCs), umbilical cord (UC-MSCs), amniotic fluid, placenta, and peripheral blood (Thomi *et al*., 2019, Basmaeil *et al*., 2020). MSCs are somatic progenitor cells (Haynesworth *et al*., 1992) differentiated from marrow hematopoietic cells based on their adherent nature in in vitro cell lines and fibroblastic morphology (Caplan, 1991). Recently, many studies have focused on MSCs, for their potential ability to migrate and mediate damage repair. MSCs facilitate neurological recovery and neo-angiogenesis via the secretion of neurotrophins and angiogenesis regulatory factors (Deng *et al*., 2016, Showalter *et al*., 2019). Furthermore, a wide number of studies has been demonstrated the potential role of MSCs in treating immune-mediated, inflammatory, and degenerative diseases (Katuchova *et al*., 2012, Staff *et al*., 2019).

Several preclinical studies explain the therapeutic effects of MSCs therapy or cell-free therapy based on MSC-derived EVs/exosomes showing the similar therapeutic outcomes of both treatments. Comparative analyses of both MSCs and their EVs illustrated different genetic cargo with the protein content contributing to various processes, including angiogenesis, adipogenesis, apoptosis, regulation of inflammation, blood coagulation, and extracellular matrix remodelling. For example, the same effect on symptoms was observed in a mouse adipose MSCs model compared with a chronic colitis mouse model. In addition, treated mice showed suppressed clinical signs and tissue damage (Heidari *et al*., 2018). Using the umbilical cord MSC-derived exosomes ameliorated clinical symptoms, reduced colonic damage, and decreased the inflammatory condition in mice with colitis when compared with the administration of MSCs (1 × 106 cells) (Ma *et al*., 2019).

The promising results obtained from preclinical studies, including BM-MCs and MSCs, in treating neurological conditions open a new door to developing non-pharmacological cell-based treatments for psychiatric disorders, especially depression. The potential therapeutic effects of adult cell-based therapies are characterised across experimental animal depression model studies (do Prado-Lima *et al*., 2019) (see Table 1). Research has verified the potential therapeutic role of MSC transplantation in depression by activating the anti-inflammatory pathways (Huang *et al*., 2020). Injection of adipose-derived mesenchymal stem cells (ADSCs) into C57BL/6 mice with chronic mild stress (CMS) decreased depressive-like behaviours, alleviated serum levels of some pro-inflammatory cytokines (CCL2, TNF-a, IL-1β, and IL-6), and increased the expression of both BDNF and its receptor in the brain tissue (Huang *et al*., 2020). In another study, implanting the encapsulated MSCs (eMSCs) into the lateral ventricle of Wistar Kyoto rats with higher depression-like behaviours and resistance to treatment against conventional antidepressant drugs showed antidepressant effects via neurogenic pathways (Kin *et al*., 2020).

**Table 1.** Several experimental and clinical studies on the effects of administering mesenchymal stem cells with different sources and exosomes in depression treatment

Preclinical studies				
	Animal model	Type of cells infused and route of administration	Major finding	Ref.
	Wistar Kyoto rats	Encapsulated MSCs (eMSCs) were implanted into the lateral ventricle of Wistar Kyoto rats	Antidepressant effects following eMSCs implantation via neurogenic pathways	(Kin *et al*., 2020)
	C57BL/6 mice	(ADSCs)	-Prevention of pro-inflammatory cytokines (CCL2, TNFa, IL-1β, and IL-6) -A higher expression of both BDNF and its receptor in the brain tissue	(Huang *et al*., 2020)
	Mice CUMS depression model	hUC-MSCs	Downregulation of pro-inflammatory genes (GFAP, Iba1, Il-1, TNF, IL-1b, and TNF-α)	(Li *et al*., 2020)
	Depression model (Corticosterone injection Sprague Dawley rats)	BMSCs-derived exosomes (100 μg/ 1 mL PBS) i.v.	Improvement in hippocampal neuron injury of depressed rats through upregulating miR-26a	(Guo *et al*., 2020)
	BALB/c mice	Exosomes derived from NK cells one time (66.42 μg i.v.)	-Decrease in depression symptoms in mice -Decreased levels of pro-inflammatory cytokines (IL-1β, IL-6, and TNF-α) released by astrocytes in vivo. -Decreased antidepressant activity in vivo experiments.	(Li *et al*., 2020)
	Two-month-old male Wistar rats	A single injection of MSCs at a dose of 1 × 106 cells/kg	MSCs alone or combined with exercise have more favourable effects in reducing depression induced by osteoarthritis.	(Abdizadeh *et al*., 2021)
	Depre			
	Flinder-Sensitive Line rats (FSLs rats), a genetic model for depression	MSC-EAAT	Elevated glutamate uptake and neurotropic factor secretion in the hippocampus lead to suppressed depressive behaviours.	(Shwartz *et al*., 2017)
	Male C57BL/6 mice	Mice received MSCs (105 cells/mouse in 0.05 mL saline) via the jugular vein	A single dose of MSCs as adjuvant therapy improved depression-like behaviour in mice that survived experimental cerebral malaria.	(Lima *et al*., 2020)
	ICR mice	Intravenous injection of mitochondria yielded from the hippocampus.	Restored ATP production Promoted neurogenesis	(Wang *et al*., 2019)
	Young (3 months old) and aged (22 months old) male rats	Plasma therapy	Increased serotonin transporter and growth-associated protein (GAP)-43	(Ghaffari-Nasab *et al*., 2022)
	Experimental animal study	Continuous and interval exercise therapy	Increased hippocampal PGC-1 α, FNDC5, and BDNF proteins were found Improved anxiety- and depression-like behaviours.	(Babaei *et al*., 2021)
	Experimental animal study on adult male C57BL/6 mice	Running wheel exercise	SIRT1 increases the PGC1a and FNDC5 levels through mediating *Bdnf* expression	(El Hayek *et al*., 2019)
Adult male C57BL/6 chronic unpredictable mild stress mice		Physical exercise therapy	Exercise improved skeletal muscle PGC-1a and BDNF levels in the hippocampus, and reduced IDO1 in skeletal muscle in stressed mice	(Dong *et al*., 2020)
	A cohort study on 49 unique prospective studies	Exercise therapy	Lower risk of developing depression in people engage with exercise	(Schuch *et al*., 2018)
	A controlled clinical trial	Exercise therapy	Improvement in quality of life and lower depression levels by aerobic and resistance exercises in women received breast cancer treatments	(Aydin *et al*., 2021)
	Prospective Cohort Studies	Exercise therapy	Physical activity provide protection against depression	(Schuch *et al*., 2018)
	A randomised clinical trial study	Programmed physical activity and antidepressant treatment	Decreased depressive symptomatology in patients who diagnosed with a mild-to-moderate depressive disorder were observed when supervised physical activity and antidepressant treatment	(Hidalgo and Sotos, 2021)
	Controlled clinical Trial	Aerobic and resistance exercises	Improvement in quality of life and lower depression were observed in woman experienced breast cancer treatments	(Aydin *et al*., 2021)
	A randomised clinical trial study	Six weeks of high-intensity interval and moderate continuous training	High-intensity interval exercise increased stress and IL-6 and moderate continuous training leads to reduced depression and TNF-α levels	(Paolucci *et al*., 2018)
		Exercise combination with monoaminergic drugs		(Martinsen, 2008)
	A randomised controlled trials	Aerobic exercise intervention	No correlation was seen between amount of exercise training and reactivity to the stress test	(Arvidson *et al*., 2020)
	A clinical trial study	Exercise therapy	Exercise-dependent elevations in BDNF serum were found in MDD patients	(Kramer *et al*., 2023)
	**Clinical findings**			
	**Target population**	**Product**	**Outcomes**	**Study**
	Treatment-resistant depressed people; (*n* = 80)	Allogeneic MSCs (108 cells single i.v)	Incidence of any treatment-emergent serious adverse events Reduced Inflammation	NCT02675556
	Treatment-resistant bipolar depression; (*n* = 30)	Allogeneic BM-SCs	Alternation in depression as assessed by the MADRS Scale.	NCT03522545
		Allogeneic MSCs (108 cells single i.v)	An incident of treatment-emergent- serious adverse events	NCT03265808
	Refractory depression; anxiety disorders; neurodegenerative diseases; (*n* = 300)	Focused ultrasound and exosomes	Beck depression inventory (BDI-II)	NCT04202770
	A randomised clinical trial on 347 patients aged ≥65 years	Physical exercise programme	The effectiveness of physical exercise was observed in treating depression	NCT03358433
	A clinical trial study	Behavioural activation (BA) plus an exercise or stretching prescription	Exercise increased serum BDNF levels	NCT02176408

**eMSCs,** encapsulated MSCs; **MSCs,** mesenchymal stem cells; **CUMS,** chronic unpredictable mild stress; **hUC-MSCs,** human umbilical cord mesenchymal stem cells; **BDNF,** brain-derived neurotrophic factor; **TrkB,** tyrosine receptor kinase; **AD-SCs,** adipose-derived mesenchymal stem cells; **FSLs,** Flinder-Sensitive Line rats; **BM-SCs,** bone marrow-derived; **FNDC5,** fibronectin type III domain-containing protein 5; **PGC1 α,** peroxisome proliferator-activated receptor γ coactiva-tor-1 α; **IDO1,** indoleamine 2,3-dioxygenase; **SIRT1,** silent information regulator 1.

The effectiveness of treatment with human umbilical cord MSCs (hUC-MSCs) was recognised for CMS in mice, along with the downregulation of pro-inflammatory genes (GFAP, Iba1, Il-1, TNF, IL-1b, and TNF-α, as well as IL-10, TGF- β, and AMPA gene expression (Li *et al*., 2020).

Moreover, the administration of BM-MCs-derived exosomes in a rat model of depression induced by corticosterone injection upregulated miR-26a levels, increased the proliferation of the hippocampus, and averted apoptosis (Guo *et al*., 2020). Administration of natural killer (NK)-cell-derived exosomes carrying miR-207 revealed the effectiveness of this method in diminishing depression symptoms in mice exposed to CMS by decreasing the levels of IL-1b, IL-6, and TNF-α release by astrocytes (Li *et al*., 2020). In a recent study, induction of osteoarthritis led to anxiety- and depression-like behaviours, and injection of MSCs alone or combined with exercise reduced depression-like symptoms (Abdizadeh *et al*., 2021). Such improvement is possibly induced by elevated glutamate uptake and neurotropic factor secretion in the hippocampus (Shwartz *et al*., 2017). According to the literature, treatment with MSCs significantly reduced depression-like behaviour in mice infected with cerebral malaria. One explanation may be due to the possible role of the MSCs, which restored BDNF and TGF-β protein levels (Lima *et al*., 2020). The clinical trials involving cell-based products or exosomes to treat depression have been listed in Table 1.

#### Mitotherapy in depression

Mitochondria in the CNS have fundamental roles, including neuroplasticity, neurotransmitter release, neurogenesis, and synaptic plasticity (Cheng *et al*., 2010). Stressful events cause the hyperactivation of HPA, leading to dysfunction in serotonergic and dopaminergic transmission and contributing to depression (Du and Pang, 2015). Serotonin potentiates mitochondrial biogenesis and bioenergetics, stimulates stress adaptation, and survives cortical neurons (Fanibunda *et al*., 2019).

Mitochondrial transport is a phenomenon by which mitochondria can be transferred from a donor cell to another (Torralba *et al*., 2016). This process has highlighted significant interest in recent years for its potential use in the treatment of mitochondrial disorders, ageing-related conditions, and other disorders (Spees *et al*., 2006).

As therapy, mitochondrial transfer has emerged as a promising option for treating a wide variety of diseases through restoring mitochondrial function, thereby reducing oxidative stress (Marcovina *et al*., 2013).

Researchers in medicine field and also biology have recently shown a great deal of interest in the importance of mitochondrial transfer. The phenomena of mitochondrial transport is associated with different mechanisms, such as gap junction channels (GJCs), extracellular vesicles (EVs), and tunneling nanotubes (TNTs) (Clemente-Suárez *et al*., 2023).

Recently the potential of mesenchymal stromal cell (MSC) mitochondrial transfer as a cell rescue strategy in regenerative medicine. Preclinical studies highlighted the MSC mitochondrial transfer ability to protect cells from mitochondrial dysfunction and oxidative stress, develop tissue repair in various disease models. The authors emphasise the importance of mitochondrial transfer in Restoring mitochondrial function is a key factor in promoting cellular health; however, suggesting the promising potential of this strategy for treating diseases, including neurodegenerative disorders, cardiovascular diseases, and muscle degeneration (Tan *et al*., 2022).

Currently, mitochondrial therapy (mitotherapy) presents a novel paradigm for neurological disorders such as AD, traumatic brain injury, cerebral stroke, spinal cord injury, and Parkinson’s disease (Hayakawa *et al*., 2016, Chien *et al*., 2018, Gollihue *et al*., 2018). Mitotherapy through the transfer of functional exogenous mitochondria into mitochondria-defective animals may prevent the disease’s progress (Javani *et al*., 2023). Evidence has shown that exogenous mitochondria can directly target animal tissue cells for therapeutic features after local and intravenous administration (Liu *et al*., 2014, Gollihue *et al*., 2018). For example, in a recent lipopolysaccharide (LPS)-induced depression model in mice, injection of exogenous mitochondria decreased anxiety and depression symptoms, neuroinflammation, oxidative stress, ATP production, and hippocampal neurogenesis (Wang *et al*., 2019). Generally, mitochondrial transplantation can alleviate depression symptoms and might be a potential treatment for inhibiting depression development.

#### Plasma therapy in depression

Depression is associated with brain responses to stress, which have a major role in the pathophysiology of late-life depression. Despite significant efforts aimed at pharmacological treatments in the elderly due to poor responses to stress-induced depression, new therapeutic approaches have been developed (Lavretsky, 2016). This poor response comes from the different aetiologies of depression in older individuals (Khundakar *et al*., 2009). In this regard, plasma obtained from young animals transferring into aged animals can decrease depression symptoms (Katsimpardi *et al*., 2014, Shytikov *et al*., 2014). For example, systematically transferring young plasma has been shown to change several age-related neuron pathologies at the molecular, functional, and cognitive levels in aged mice (Villeda *et al*., 2014). The antidepressant effect of young plasma in old age is due to significantly increased serotonin transporter levels and growth-associated protein (GAP)-43 following chronic administration of young plasma (Ghaffari-Nasab *et al*., 2022).

#### Exercise therapy in depression

Studies have shown that, as a non-pharmacological method, exercise can help reduce symptoms of depression with effectiveness comparable to drug therapy and other psychological approaches (Blumenthal and Doraiswamy, 2014, Harvey *et al*., 2018). In addition, exercise can also prevent other diseases, including obesity, diabetes, and osteoporosis (Qaseem *et al*., 2017, Kumar *et al*., 2019). Numerous studies have demonstrated the benefits of exercise and physical activity for enhancing mood and avoiding mood disorders. A meta-analysis of observational data suggests that young people who engage in regular physical activity are at lower risk of developing depression (Schuch *et al*., 2018). Hidalgo et al., showed that (Hidalgo and Sotos, 2021) antidepressant medication and physical exercise both had comparable benefits in treating depression, although the adverse effects of antidepressant medication were greater. This was a randomised controlled trial that included 347 people over 65 years old with depression. The results of the study showed that after one month, the cumulative incidence of depression was similar in both groups. In the following months, the proportion of recovered patients was higher in the antidepressant treatment group, although the side effects were also relatively higher in this group. Exercise can also increase a person’s sense of optimism, purpose, and meaning and help deal with stress. In addition, exercise can enhance social support because it gives people the opportunity to interact with others, especially when done in a group setting such as cardiac rehabilitation programmes. The improvement in mood that may follow from starting to exercise is most likely due to a synergistic combination of psychological, biological, and social pathways stimulated by regular exercise and physical activity. Aydin and colleagues (Aydin *et al*., 2021) demonstrated that both resistance and aerobic exercises enhanced quality of life and reduced depression symptoms in women who had previously received breast cancer therapies. A growing body of research indicates that both exercise and antidepressant drugs may reduce depression via similar neuromolecular processes, such as increased availability of NE and serotonin, regulation of HPA axis activity (Arvidson *et al*., 2020), increased expression of neurotrophic factors (i.e. BDNF) (NCT02176408), and decreased systemic inflammatory signalling (Mathur and Pedersen, 2008). These processes influence the development of new neurons, increase synaptic connections between neurons, and increase cerebral vasculature (Voss *et al*., 2013, Erickson, Leckie and Weinstein 2014).

Exercise, interestingly may serve as a non-pharmacological alternative approach in combination with monoaminergic drugs for cognitive symptoms in MDD (Martinsen, 2008). Likewise, (Paolucci *et al*., 2018) found that after six weeks of high-intensity interval exercise increased stress and IL-6 and moderate continuous training leads to reduced depression and TNF-α levels. According to the results of a clinical trial conducted in 2023, patients with MDD who did heavy exercise had significantly higher BDNF levels than the group who did moderate activity (Kramer *et al*., 2023). Furthermore, due to elevation in 5-HT level by upregulating peroxisome proliferator-activated receptor γ coactiva-tor-1 α (PGC-1 α), reducing oxidative stress and the inflammatory response, decreasing indoleamine 2,3-dioxygenase (IDO) activity, and increasing TRY activity, exercise can exert an antidepressant effect (Dong *et al*., 2020). In adittion, the expression of PGC-1 α, which is dependent to fibronectin type III domain-containing protein 5 (FNDC5) increases and results in the expression of BDNF, a protective factor that can regulate FNDC5 gene expression (Babaei *et al*., 2021). In other words, exercise can ameliorate depression by upregulating PGC-1 α, FNDC5, and BDNF expression. In another study, (El Hayek *et al*., 2019) reported that exercise through release of lactic acid can activate SIRT1 resulting in upregulation of BDNF levels in the hippocampus through the PGC-1α/FNDC5 pathway, supporting the neuroprotective role of BDNF. Generally, exercise has been shown to have therapeutic benefits for depression thus far. Ensuring the efficacy of exercise programmes can be facilitated by selecting the appropriate activity for the individual and providing long-term follow-up. However, further research with larger sample sizes is needed to evaluate and prove this issue, as well as to understand the biological relationships.

#### TMS therapy in depression

A successful candidate for MDD patients who are resistance to therapy is TMS, as a non-invasive technique, in which stimulation of nerve cells carried out with no requirement to craniotomy or seizure induction to (George *et al*., 2013). This noninvasive method involves the application of a magnetic field as it is changed rapidly and applied to the superficial layers of the cerebral cortex, which acts as a secondary coil in this situation (Horvath *et al*., 2011).

#### Transcranial direct current stimulation (tDCS) therapy in depression

Transcranial direct current stimulation (tDCS) is among the noninvasive therapies in which brain stimulation have emerged as relevant therapies. According to knowledge on specific brain areas involved in psychiatric diseases tDCS seems to be promising due to its cost and easily using (Lefaucheur *et al*., 2017). Bidirectional alternations of postsynaptic connections appears to be mediate this sustainable effects similar to NMDA-related mechanisms occurred in long-term potentiation and depression (Das *et al*., 2016). Given the implication of alterations of neuroplasticity in pathologic condition such as psychiatric disorders, tDCS might be a promising therapeutic alternative to treatment of such pathological plasticity modification (Kuo *et al*., 2014). A large number of literature stablished the beneficial effects of tDCS for treating mainly (mainly depression, neurological diseases, and other psychiatric disorders (Mondino *et al*., 2014).

**Figure 2.**
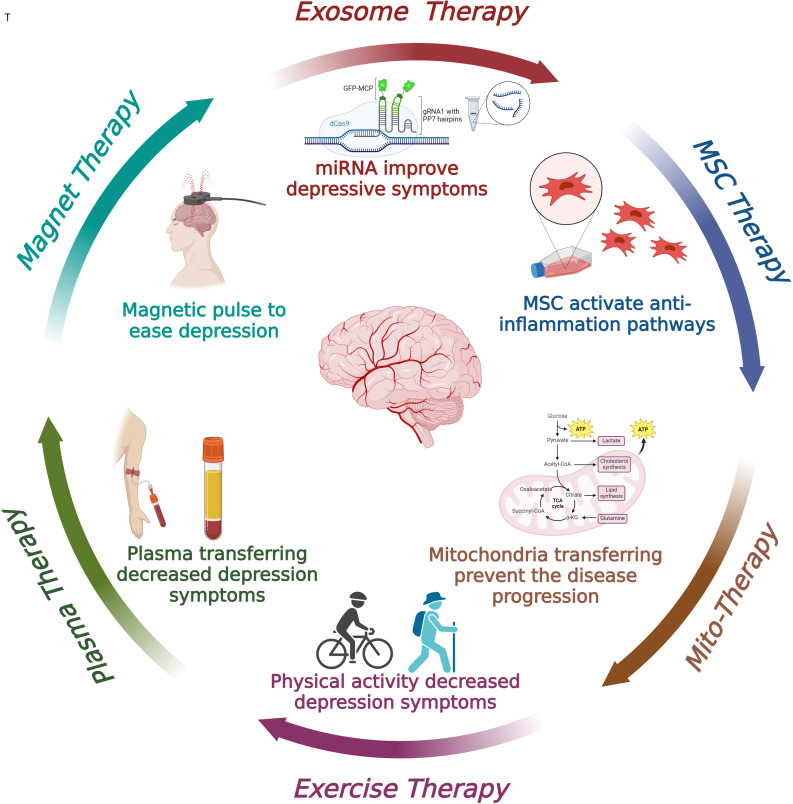
Non-pharmacological therapeutic features for depression. MSC: mesenchymal stem cell.

As shown in Figure 2, non-pharmacological therapeutic approaches have been illustrated.

Considering data obtained from experimental and human studies, it was revealed that chronic stress induced depression is associated with hyper activation of immunological processes, suggesting a potential role of neuroinflammatory biomarkers underlying depression, as is the case with other psychiatric illnesses. Regardless of the large number of effective anxiolytic treatments for this mental disorder, a substantial number of patients have limited benefit from common pharmacological treatment. Despite the fact that noninvasive therapies cannot currently target depression-related underlying mechanisms directly, cell-based therapies are being investigated as a novel method for indirectly targeting downstream steps in the brain. For instance, transferring the content of exosomes, which are composed of various clinically critical biomolecules, could potentially affect intercellular communication in various physiological or pathological conditions (Yuyama and Igarashi, 2016).

We hope that with increasing containment of the pandemic, a more systematic investigation of these cases will be possible. As proposed in the field of EV-based, MSCs-based, mitotherapy, and plasma therapy strategies, it would be very helpful to shed light on defining novel approaches to relevant advances in depression therapy. Despite the limited number of preclinical studies, considering the various effective cell-based approaches to treating depression, further research must be explored. Nevertheless, the approaches discussed above can provide new advances in treatment and can be more effective methods for treating depression.

A large number of studies have demonstrated that miRNAs contribute to synaptic plasticity, which is linked to the development of depression (Zhou *et al*., 2021). Furthermore, miRNAs can cross the BBB and enter the CNS using exosomes as carriers to suppress various physiological functions, such as neurogenesis, neurotransmission, synaptic morphology, structure, and synaptic energy metabolism (Gao *et al*., 2022). However, further studies are needed to evaluate whether miRNA dysregulation or a combination of miRNAs are involved in the pathology of depression or represent a potential marker for treatment response (Li *et al*., 2021). In other words, miRNAs may be used as novel medications or as a new biomarker for the diagnosis of depression or therapeutic targets. BMSC-derived exosomes, through upregulating miR-26a, improve hippocampal neuronal injury in depressed rats (Guo *et al*., 2020). In addition, data from normal mouse exosomes indicate that overexpressed miR207 inhibits the NF-κB signalling pathway in astrocytes and reduces the production of pro-inflammatory factors, which results in alleviating depressive symptoms in the depressed-mice model (Li *et al*., 2020). Also, the application of plasma exosomes represents antidepressant-like effects on sigma-1 receptors induced by LPS in an animal model (Bhatt *et al*., 2021, Wang *et al*., 2021). Therefore, further studies can develop new treatments on the basis of exosomes for depression. A number of preclinical studies explain the therapeutic effects of MSCs therapy, demonstrating the same therapeutic consequences for both treatments (Heidari *et al*., 2018). Although several clinical trials according to the application of MSCs for depression therapy have emerged or are developing, exosomes derived from MSCs have become a promising approach for treating mood disorders such as depression. In this case, cell therapy via BM-MCs or MSCs transplantation or the participation of cell products such as exosomes in such treatments may have a potent place in depression, which is resistant to common therapies. Therapeutic effects of exosomes also introduce a novel strategy for neurodegenerative diseases (Xiong *et al*., 2017). Due to the limited number of preclinical studies, additional clinical trial studies are needed. Finally, mitotherapy through transferring functional exogenous mitochondria into mitochondria-defective animals (Javani *et al*., 2023) or plasma therapy (Katsimpardi *et al*., 2014, Shytikov *et al*., 2014) are the other novel non-pharmacological methods for depression that may prevent the disease’s progress.

Furthermore, good tolerability of non-invasiveness of tDCS and TMS which introduce a successful option for MDD patients with therapy resistance support the need for additional research into depression therapy.

## Discussion

Considering data obtained from experimental and human studies, it was revealed that chronic stress induced depression is associated with hyper activation of immunological processes, suggesting a potential role of neuroinflammatory biomarkers underlying depression, as is the case with other psychiatric illnesses. Regardless of the large number of effective anxiolytic treatments for this mental disorder, a substantial number of patients have limited benefit from common pharmacological treatment. Despite the fact that noninvasive therapies cannot currently target depression-related underlying mechanisms directly, cell-based therapies are being investigated as a novel method for indirectly targeting downstream steps in the brain. For instance, transferring the content of exosomes, which are composed of various clinically critical biomolecules, could potentially affect intercellular communication in various physiological or pathological conditions (Yuyama and Igarashi, 2016).

A large number of studies have demonstrated that miRNAs contribute to synaptic plasticity, which is linked to the development of depression (Zhou *et al*., 2021). Furthermore, miRNAs can cross the BBB and enter the CNS using exosomes as carriers to suppress various physiological functions, such as neurogenesis, neurotransmission, synaptic morphology, structure, and synaptic energy metabolism (Gao *et al*., 2022). Because of the issue of whether miRNA dysregulation or a combination of miRNAs are involved in the pathology of depression or represent a potential marker for treatment response, further studies are needed to evaluate that (Li *et al*., 2021). In other words, miRNAs may be used as novel medications or as a new biomarker for the diagnosis of depression or therapeutic targets. BMSC-derived exosomes, through upregulating miR-26a, improve hippocampal neuronal injury in depressed rats (Guo *et al*., 2020). In addition, data from normal mouse exosomes indicates that overexpressed miR207 inhibits the NF-κB signalling pathway in astrocytes and reduces the production of pro-inflammatory factors, which results in alleviating depressive symptoms in the depressed-mice model (Li *et al*., 2020). Also, the application of plasma exosomes represents antidepressant-like effects on sigma-1 receptors induced by LPS in an animal model (Bhatt *et al*., 2021, Wang *et al*., 2021). Therefore, further studies can develop new treatments on the basis of exosomes for depression. A number of preclinical studies explain the therapeutic effects of MSCs therapy, demonstrating the same therapeutic consequences for both treatments (Heidari *et al*., 2018). Although several clinical trials according to the application of MSCs for depression therapy have emerged or are developing, exosomes derived from MSCs have become a promising approach for treating mood disorders such as depression. In this case, cell therapy via BM-MCs or MSCs transplantation or the participation of cell products such as exosomes in such treatments may have a potent place in depression, which is resistant to common therapies. Therapeutic effects of exosomes also introduce a novel strategy for neurodegenerative diseases (Xiong *et al*., 2017). Due to the limited number of preclinical studies, additional clinical trial studies are needed.

Mitotherapy through transferring functional exogenous mitochondria into mitochondria-defective animals (Javani *et al*., 2023) or plasma therapy (Katsimpardi *et al*., 2014, Shytikov *et al*., 2014) are the other novel non-pharmacological methods for depression that may prevent the disease’s progress.

Furthermore, good tolerability of non-invasiveness of tDCS and TMS which introduce a successful option for MDD patients with therapy resistance support the need for additional research into depression therapy.

## Conclusion and future perspective

Understanding the neurobiology of MDD will enable the use of more effective treatments, resulting in significant improvements in clinical features and patients’ quality of life. So sustained efforts related to non-pharmacological treatments for representing a promising new target need for develop. Non-pharmacological therapies, have garnered a great deal of attention. Despite TMS and DBS techniques being considered to affect mitochondrial function through promoting neuroplasticity for patients with TRD, identifying more precise selection criteria to predict optimise outcomes remains unclear. Additionally, due to the involvement of mitochondrial dysfunction in the pathogenesis of MDD, focusing on biomarkers related to mitochondrial activity may be helpful in detecting and prognosing of TRD patients. This effort opens new avenue for creating more specific therapeutic approaches.

Due to the heterogenic status of MDD, the pathological and pharmacological mechanisms of this mental disorder are still unclear, and novel therapeutic strategies for MDD are limited. In clinic, SSRIs are known as the first-line treatments for MDD; however, more patients don not represent a well response to the antidepressants which are currently available. In this regard, more research on the pharmacological mechanisms of MDD is still needed.

A number of cellular and molecular alternations in CNS are connected to the MDD. Therefore, understanding the pathophysiology and therapeutic issues have considered for further research. We hope that with increasing attention to non-pharmacological therapeutic methods, a more systematic investigation of these cases will be possible. As proposed in the field of EV-based, MSCs-based, mitotherapy, and plasma therapy strategies, it would be very helpful to shed light on defining novel approaches to relevant advances in depression therapy. In the future, EVs may be used as effective treatment for identification of CNS-related disorders especially for depression.

By development of cell-based therapies especially the technology of cell-based products, trying of all aspects of such therapies may be the best alternative as an antidepressant treatment, with lower costs and the more established method than the routines. The existence of the challenges as a consequences of resistant depression treatment, the possibility and feasibility of these procedures as an alternative treatment must be explored.

In addition, according to the high prevalence of MDD worldwide, the prevent of its occurrence is crucial. Lifestyle interventions such as exercise is an evolving medical specialty that aims to prevent chronic diseases. Recently, many studies have been focused on the occurrence and ameliorating depressive symptoms in MDD patients which prevented by lifestyle medicine such as exercise. As highlighted above, the use of cell-based approaches to treating depression is still restricted by the limited current preclinical studies. However, the lack of such studies might be explained. Nevertheless, the approaches discussed above can provide new advances in treatment and can be more effective methods.

Although mitochondrial transplantation is somehow promising for the targeted treatment of MDD, the successful application of this technique in laboratory experiments and clinical practice needs to more studies. Processing the mitochondria outside the body and reintroduced into the same patient provides high specificity and makes this therapy with less post-transplantation immune rejection. While results obtained from experimental models have presented potential therapeutic effect of stem cells in depression therapy, gaps remain to be explored. Aditional studies are needed to discuss the safety of any MSCs from donors who have a different genetic background than the patient as an antidepressant therapy. Moreover, gaps such as the optimal dose, administration route, and fundamental mechanisms of action are needed to establish in further studies.

In summary, ongoing research into the neurobiology and treatment of MDD has the potential to radically transform clinical care and improve outcomes for patients. However, further studies are needed to translate these advances into effective and personalised therapeutic strategies that address the complex interactions between the neurobiological systems implicated in MDD.
